# Evaluation of Biological Value and Appraisal of Polyphenols and Glucosinolates from Organic Baby-Leaf Salads as Antioxidants and Antimicrobials against Important Human Pathogenic Bacteria

**DOI:** 10.3390/molecules18044651

**Published:** 2013-04-19

**Authors:** Alfredo Aires, Esperança Marques, Rosa Carvalho, Eduardo A. S. Rosa, Maria J. Saavedra

**Affiliations:** 1 CITAB—Centre for the Research and Technology for Agro-Environment and Biological Sciences, Universidade de Trás-os-Montes e Alto Douro, Apartado 1013, 5000-801 Vila Real, Portugal; E-Mail: erosa@utad.pt; 2 Agronomy Department, Universidade de Trás-os-Montes e Alto Douro, Apartado 1013, 5000-801 Vila Real, Portugal;E-Mail: eferreiramarques@sapo.pt (E.M.); rpaula@utad.pt (R.C.); 3 CECAV—Veterinary and Animal Science Research Center, Universidade de Trás-os-Montes e Alto Douro, Apartado 1013, 5000-801 Vila Real, Portugal; E-Mail: saavedra@utad.pt

**Keywords:** baby-leaf salads, phytochemicals, polyphenols, glucosinolates, antioxidants, antimicrobials

## Abstract

The present investigation has been carried out to investigate the biological role of four different types of baby-leaf salads and to study their potential as natural sources of antioxidants and antimicrobials against several isolates from important human pathogenic bacteria. Four single types of salads (green lettuce, red lettuce, rucola and watercress) and two mixtures [(1) red lettuce+green lettuce; (2) green lettuce + red lettuce + watercress + rucola] were assayed. The HPLC analysis revealed interesting levels of polyphenols and glucosinolates. The results showed a significant variation (*p* < 0.05) of polyphenols and glucosinolates with plant material. Nine different types of polyphenols grouped in three major classes were found: gallic acid, chlorogenic acid, caffeic acid and dicaffeoyltartaric acid (phenolic acids); quercitin-3-*O*-rutinoside, quercitin-3-*O*-rhamnoside, luteolin-7-*O*-glucoside and isorhamnetin (flavonoids); and cyanidin-3-glucoside (anthocyanins). Only three different glucosinolates were found: glucoraphanin; gluconasturtiin and 4-methoxy-glucobrassicin. A positive correlation was detected between polyphenol contents and antioxidant activity. Red lettuce and mixture 1 were the baby-leaf salads with the highest antioxidant potential. As for the antimicrobial activity, the results showed a selective effect of chemicals against Gram-positive and Gram-negative bacteria and *Enterococcus faecalis* and *Staphylococcus aureus* were the bacteria most affected by the phytochemicals. Based on the results achieved baby-leaf salads represent an important source of natural antioxidants and antimicrobial substances.

## 1. Introduction

Fruits and vegetables are rich in vitamins and minerals. They also contain other natural compounds which are derived from plant secondary metabolism and thus named secondary plant metabolites. These compounds are important, not only due to their role in plant defence mechanisms against pathogens and diseases [1], but also because their recognized antioxidant, antimicrobial and anticarcinogenic properties [2]. Several epidemiological studies have revealed a direct relation between the consumption of vegetables and decreasing of hypertension, diabetes and obesity [3]. Moreover, numerous studies indicates that antioxidants found at high levels in vegetables and fruits can protect the human body from free radicals and retard the progress of many chronic diseases [4]. These compounds include carotenoids, ascorbic acid, phenolics and among them the flavonoids are a potent *in vitro* antioxidant group [5]. The global production and consumption of fresh baby-leaf vegetables has greatly expanded in recent years [6] as result of the recognition of the health-promoting potential of various compounds present in this type of plant material [7]. These trends have led to the development of advanced industrial technologies that provide consumers with several types of salads, vegetables, fruits and juices [8,9]. The demand for high quality ready-to-eat fruits and baby-leaf salads has been rapidly growing in recent years, because they are convenient and nutritious snack alternatives [10], despite being generally more fragile than the mature fresh raw materials. Physical damage during the cutting processes is often a cause of increasing respiration rates, biochemical changes and microbial spoilage, which often result in the degradation of color, texture and flavor and microbial growth [11,12]. Concerning the changes of polyphenols and other bioactive compounds and antioxidant capacity in baby-leaf salads, little information is available. As part of a continuing investigation for screening biological and healthy compounds, scientific investigations to determine the therapeutic potential of this transient and fragile type of plant material are still limited. Moreover, there is a lack of information about their potential effect in human bacteria microbiota. No systematic information, until now, has been published about the antibacterial activity of this plant material type when incorporated daily in the human diet. Their effect on pathogenic bacteria and what are the most important phytochemicals to gastrointestinal segment bacteria are questions that still need to be answered. Therefore, the aim of the present study was to evaluate the phytochemical composition, to identify and characterize the antioxidant and the antibacterial activities (against Gram-positive and Gram-negative pathogenic bacteria from food, animal and human source), in four different baby-leaf salads (green lettuce, red lettuce, watercress and rucola) consumed alone or in mixtures. Finally, we intend to enhance the scientific knowledge regarding the quality, safety and phytochemical composition, and the antioxidant and antibacterial effects of these vegetables against pathogenic bacteria. 

## 2. Results and Discussion

### 2.1. Total Phenolic Compounds

The average TPC levels are presented in Table 1. The results showed that red lettuce was the plant material with the highest TP content, followed by the mixture 1 and the remaining baby-leaf salads. Green lettuce was the baby-leaf with the lowest average levels of TPC. Also, the results showed that the differences in average levels of TP among the different type of salads were significantly different (*p* < 0.05), which means that eating red lettuce has a different effect than eating green lettuce. In average, the TPC value in red lettuce was three times higher than the TPC present in rucola and watercress and four times higher than the TPC in green lettuce and mixture 2 (Table 1). Within the mixtures, the mixture 1 (green lettuce + red lettuce) revealed the highest TPC value (Table 1), which could be attributed to the contribution of red lettuce. The lower TPC in mixture 2 (green lettuce + red lettuce + watercress + rucola) could be due to a suppressive effect rather than an additive one of the different samples. In fact, for mixture 1 the average level of TPC (350.9 mg Gallic Acid Equivalent (GAE)·100 g^−1^ DW) was higher than the average of the arithmetic sum between green lettuce and red lettuce (331.7 mg GAE·100 g^−1^ DW), whilst for mixture 2 the average level of TPC (137.0 mg GAE·100 g^−1^ DW), which was lower than the average of the arithmetic sum between green lettuce, red lettuce, watercress and rucola (252.9 mg GAE·100 g^−1^ DW). Not always the final effect of mixture represents the sum of the isolate effect of the parts.

**Table 1:** Total phenolic compounds of the baby-leaf salads samples ^1,2^.

Samples	Average levels (mg of gallic acid equivalent (GAE)·100 g^−1^ DW)
Green lettuce	131.4 ± 7.9 e
Red lettuce	532.0 ± 14.5 a
Rucola	161.3 ± 9.0 cd
Watercress	186.9 ± 3.5 c
Mixture 1	350.9 ± 12.1 b
Mixture 2	137.0 ± 3.1 de

^1^ Values are expressed as mean ± SEM (standard error of the mean) of three replicates. ^2^ Numbers with different letters in the same column are significantly different (*p* < 0.05), accordingly to the Duncan test.

### 2.2. Individual Phenolics and Glucosinolates

In the present study nine different phenolics were identified divided in three different classes: phenolic acids, flavonoids and anthocyanins (Table 2).

**Table 2:** Average levels of individual phenolics quantified in baby-leaf salads samples by HPLC, expressed as mg·100 g^−1^ DW ^1,2,3^.

Polyphenols	Samples					
	Green lettuce	Red lettuce	Rucola	Watercress	Mixture 1 (green lettuce + red lettuce)	Mixture 2 (green lettuce + red lettuce + watercress + rucola)
Gallic acid	n.d.	n.d.	n.d.	1.58 ± 0.18 ^c^	n.d.	1.15 ± 0.034 ^d^
Chlorogenic acid	2.24 ± 0.02 ^d^	5.97 ± 0.38 ^f^	n.d.	3.25 ± 0.33 ^b^	6.88 ± 0.17 ^e^	1.12 ± 0.014 ^d^
Caffeic acid	9.26 ± 0.93 ^c^	27.54 ± 0.51 ^d^	0.17 ± 0.0 ^d^	0.17 ± 0.0 ^d^	23.17 ± 0.55 ^c^	0.17 ± 0.0 ^e^
Caffeic derivatives	14.32 ± 0.74 ^b^	52.44 ± 1.25 ^b^	n.d.	5.48 ± 1.54 ^b^	59.27 ± 1.25 ^b^	8.22 ± 0.35 ^b^
Cyanidin-3-Glucoside	0.062 ± 0.0 ^e^	31.20 ± 0.74 ^c^	0.062 ± 0.0 ^d^	0.062 ± 0.0 ^d^	21.91 ± 1.33 ^c^	3.04 ± 0.069 ^c^
Quercetin-3-*O*-rutinoside	7.08 ± 0.39 ^c^	10.93 ± 0.57 ^e^	10.06 ± 0.22 ^c^	19.95 ± 0.16 ^a^	25.92 ± 1.42 ^c^	4.36 ± 0.28 ^c^
Quercetin-3-*O*-rhamnoside	n.d.	114.41 ± 1.86 ^a^	115.58 ± 2.44 ^a^	n.d.	128.93 ± 5.09 ^a^	n.d.
Luteolin-4-*O*-glucoside	6.36 ± 0.19 ^d^	21.68 ± 0.92 ^d^	n.d.	n.d.	13.64 ± 2.36 ^d^	0.76 ± 0.07 ^e^
Isorhamnetin	18.16 ± 0.17 ^a^	10.67 ± 0.65 ^c^	23.15 ± 0.67 ^b^	19.32 ± 0.11 ^a^	12.34 ± 1.43 ^d^	40.56 ± 2.23 ^a^

^1^ Values are expressed as mean ± SEM (standard error of the mean) of three replicates. ^2^ Numbers in the same column with different letters are significantly different (*p* < 0.05), accordingly to Duncan test. ^3^ n.d. — not detected.

**Figure 1:**
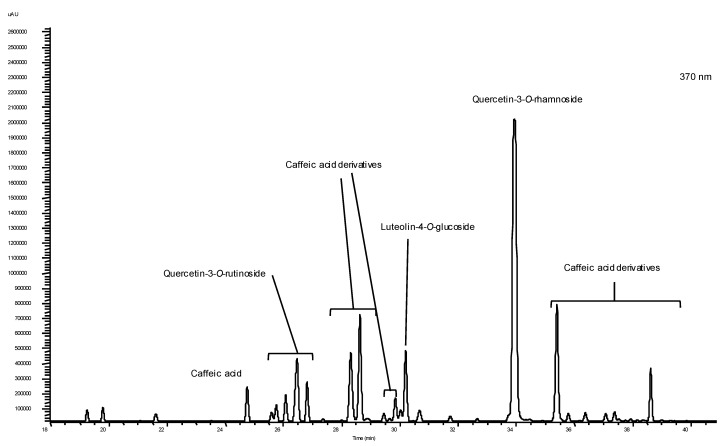
Example of a HPLC chromatogram of polyphenols detected in a red lettuce baby-leaf extract recorded at 370 nm.

The HPLC chromatograms of baby-leaf extracts recorded at 280, 320, 370 and 520 nm, were characterized by the presence of isorhamnetin, quercitin-3-*O*-rutinoside, cyanidin-3-glucoside and caffeic acid and caffeic acid isomers/derivatives. Chlorogenic acid, luteolin-4-*O*-glucoside and quercitin-3-*O*-rhamnoside were quantified only in four extracts and gallic acid was only quantified in two extracts. Ferulic acid and luteolin-7-*O*-glucoside were detected under the limit of quantification in all extracts studied, thus no values are presented (Table 2). The UV spectra detected and one example of chromatograms obtained in this study is presented in the Figure 1. The statistical analysis revealed that phenolic composition was significantly dependent on plant sample (*p* < 0.05). Green lettuce and watercress presented higher contents of phenolic acids, namely chlorogenic and caffeic acids, whilst red lettuce and rucola showed higher contents of flavonoids, namely quercetin compounds. Cyanidine-3-glucoside was detected in significant amounts in red lettuce, whilst green lettuce, rucola and watercress showed vestigial levels (Table 2). These differences indicate that profiles and average levels of polyphenols could be an intrinsic characteristic of type of vegetable analysed, which means that, in terms of phenolic profile, eating red lettuce could have a different biological effect than eating watercress or even green lettuce. Within the mixtures, the mixture 1 presented similar diversity to the red lettuce. Mixture 2 presented lower average levels in individual phenolics, contrary to expectations. In concordance to the previous results in TPC by Folin-Ciocalteau assay, mixture 2 didn't have an additive effect. This decrease could be caused by some changes in the mixture matrices which could explain the lower average levels of individual phenolics in mixtures than in individual samples. 

**Figure 2:**
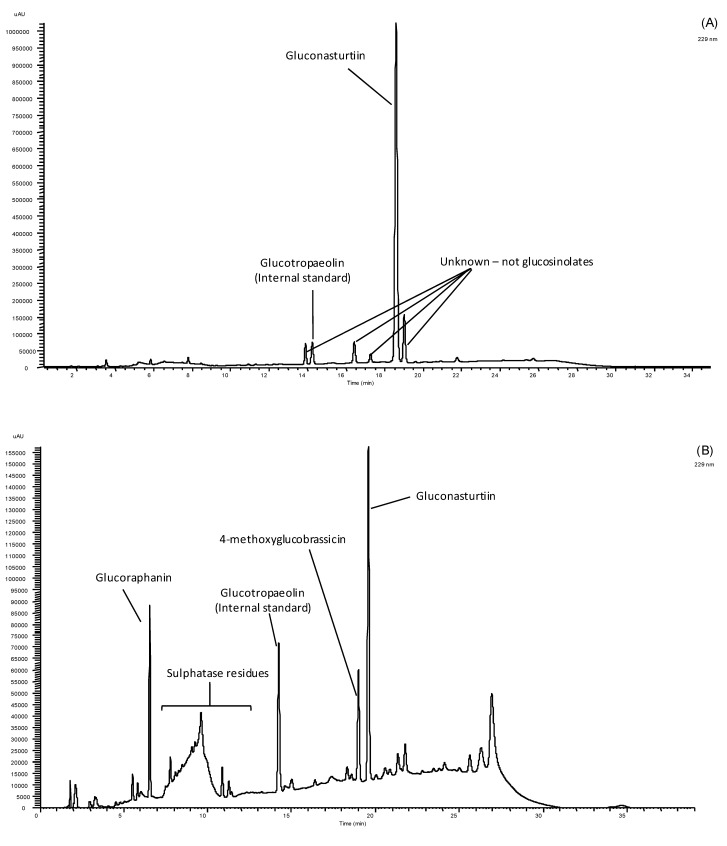
HPLC chromatogram of glucosinolates detected in a watercress (**A**) and rucola (**B**) baby-leaf extract recorded at 229 nm.

In watercress and rucola samples three different GSs were identified: glucoraphanin, gluconasturtiin and 4-methoxyglucobrassicin, as shown by the corresponding HPLC chromatogram (Figure 2). 

The levels of individual GSs (Table 3) varied significantly (*p* < 0.05) with plant extract, suggesting that the type of vegetable can affect the quantity and type of GSs present and therefore affecting the biological role of salads. It seems that, from a human health point of view, watercress has more biological interest, because it provides higher average levels of total GSs, in particularly gluconasturtiin, the precursor of 2-phenylethylisothiocyanate, one of the more biologically active GS hydrolysis products [3]. Nevertheless, rucola salad is also important, but by the diversity of GS and due to the moderate content of glucoraphanin, the precursor of the isothiocyanate 4-methylsulfinyl- butylisothiocyanate, also known as sulforaphane, one of the most potent inhibitors of the early stages of carcinogenetic processes via inhibition of phase I enzymes and induction of phase II enzymes [3,13,14]. 

**Table 3:** Average GSs content quantified by HPLC in baby-leaf salads samples, expressed as μmol.100 g^−1^ of DW ^1,2,3^.

Samples	Glucoraphanin	Gluconasturtiin	4-Methoxyglucobrassicin	Total GSs
Watercress	n.d.	3143.19 ± 202.76 a	n.d.	3143.19 ± 202.76 a
Rucola	238.28 ± 43.64	144.95 ± 3.95 b	396.59 ± 43.95	779.81 ± 11.36 b

^1^ Values are expressed as mean ± SEM (standard error of the mean) of three replicates. ^2^ Numbers with different letters in the same column are significantly different (*p* < 0.05), accordingly to Duncan test. ^3^ n.d. — not detected.

### 2.3. Antioxidant Activity

The antioxidant activity of the samples was expressed as DPPH scavenging activity (%) and as IC_50_ values (mg·mL^−1^). Results can be seen in Table 4 and varied in % of DPPH scavenging activity between 81.67 ± 1.22% and 7.96 ± 3.53% (Table 4). Only red lettuce and mixture 1 have similar AA values. The Duncan test shown a significant variation (*p* < 0.05) of AA with salad sample (Table 4). Our average values of AA for these baby-leaf salads were higher, when compared with the AA exhibited by other type of baby-leaf salads, fresh and cooked vegetables, namely spinach, broccoli, and tomato [15,16] which are frequently used as antioxidant indicator crops.

**Table 4:** Average levels of antioxidant activity of methanolic extracts (1.0 mg·mL^−1^) from baby-leaf salads samples, and IC_50_ average values ^1,2^.

Samples	% DPPH• scavenging activity	IC_50_(mg·mL^−1^)
Green lettuce	41.0 ± 6.66 d	1.43 ± 0.03 bc
Red lettuce	79.7 ± 1.38 b	0.23 ± 0.03 d
Rucola	8.0 ± 3.53 f	3.03 ± 0.58 a
Watercress	25.4 ± 2.46 e	1.87 ± 0.03 b
Mixture 1 (green lettuce + red lettuce)	81.7 ± 1.22 b	0.37 ± 0.03 d
Mixture 2 (green lettuce + red lettuce + watercress + rucola)	73.8 ± 0.12 c	0.70 ± 0.00 cd
Trolox (positive control)	83.3 ± 0.00 a	

^1^ Values are expressed as mean ± SEM (standard error of the mean) of three replicates. ^2^ Numbers with different letters in the same column are significantly different (*p* < 0.05), accordingly to Duncan test.

It was noted that the high AA presented by mixture 1 (green lettuce + red lettuce) might be attributed to the mixture of these two type of lettuce, being this contribution additive. In fact the average level of AA for mixture 1 (81.7%) was higher than the average of the arithmetic sum between green lettuce and red lettuce (60.4%), which could mean that compounds in green and red lettuce act synergistically. A similar tendency was noted for mixture 2, where the average level of AA (73.8%) was higher than the average of the arithmetic sum between green lettuce, red lettuce, watercress and rucola (38.5%). As in mixture 1, in more complex mixtures the several compounds related with antioxidant activity act synergistically. In this case the AA observed could be due to not only phenolics, but also from the presence of other phytochemicals, such as ascorbic acid, tocopherol and pigments (chlorophylls a, b) as well as the synergistic effects among them, which may also contribute to the increased AA values. 

As we expected, Trolox (an analog of vitamin E) was more efficient at scavenging DPPH radicals than all the samples. Nevertheless, the red lettuce and the mixture 1 presented similar average values to those presented by Trolox (Table 4). In relation to the IC_50_ average values, the results achieved are in agreement with the AA values. The baby leaf samples with lowest EC_50_ value and therefore high AA were red lettuce and mixture 1.

**Figure 3:**
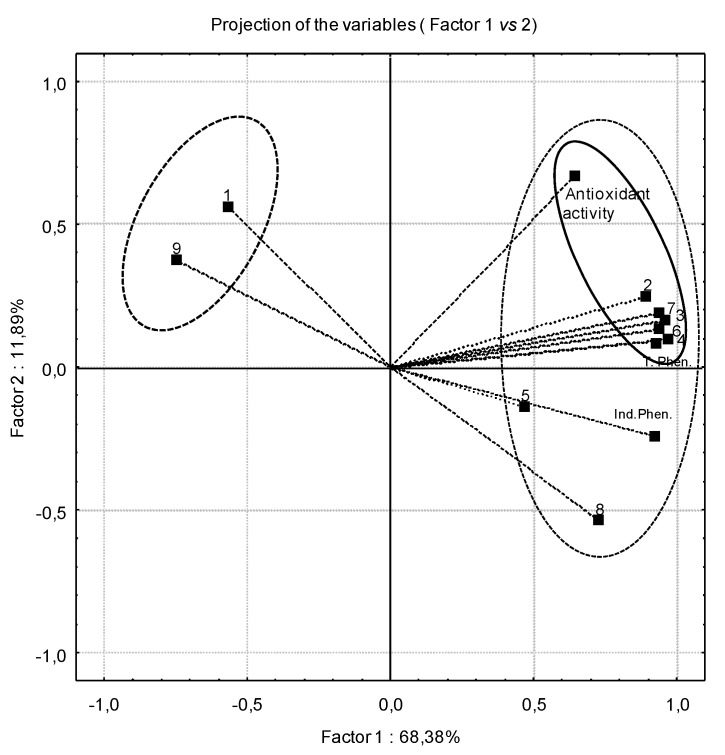
Multivariable analysis results: (**1**) gallic acid (**2**) chlorogenic acid; (**3**) caffeic acid; (**4**) cyanidin-3-glucoside; (**5**) quercetin-3-*O*-rutinoside; (**6**) Luteolin-4-*O*-glucoside; (**7**) caffeic derivatives; (8) quercetin-3-*O*-rhamnoside; (9) Isorhamnetin.

To evaluate the eventual correlation between TPC, individual phenolics and AA, we applied the Pearson correlation (Table 5) and PCA analysis (Figure 3). These two results showed an association between the increase of AA and increased levels of phenolic compounds. The correlation between GSs and AA were not evaluated because the GSs detected in the current samples are not associated to AA, as shown by previous studies [17]. 

**Table 5:** Coefficients and degrees of significance of Pearson correlations between the different parameters ^1^.

	Total phenolic content	Total individual phenolics	% DPPH• (at 1 mg·mL^−1^)
Total phenolic content	1.000	0.855 **	0.599 **
Gallic acid	−0.394	−0.641	−0.133
Chlorogenic acid	0.816 **	0.724 **	0.669 **
Caffeic acid	0.922 **	0.844 **	0.687 **
Caffeic acid derivatives	0.812 **	0.772 **	0.739 **
Cyanidine-3-glucoside	0.971 **	0.889 **	0.736 **
Quercitin-3-*O*-rutinoside	0.338	0.463	0.111
Luteolin-7-*O*-glucoside	0.913 **	0.799 **	0.680 **
Quercitin-3-*O*-rhamnoside	0.663 **	0.923 **	0.194
Isorhamnetin	−0.658	−0.638	−0.079
Total individual phenolics	0.855 **	1.000	0.495
% DPPH at 1 mg·mL^−1^	0.599 **	0.495	1.000

^1^ Correlation is significant when *p* < 0.01 (numbers with **).

Based on the results, it seems that antioxidant activity in these samples is related with phenolic compounds content***.*** The higher AA of red lettuce and mixture 1 may be attributed to the high average levels of total and individual phenolics, caffeic acid, cyanidin-3-glucoside, quercitin-3-*O*-rhamnoside, quercitin-3-*O*-rutinoside and luteolin-7-*O*-glucoside, which have been associated with higher AA values in several vegetables matrices [18].

### 2.4. Antimicrobial Activity

The antimicrobial activity of baby-leaf samples and pure compounds is presented in Table 6. The results, in general, showed an absence of antibacterial effect of the salads extracts, except for the isolate *Enterococcus faecalis* 49 (Gram positive bacteria strain of animal origin). For this isolate we verified an inhibition effect (moderate, +) for all extract samples, especially for the extract of rucola (Table 6). Nevertheless, the inhibition zones recorded for baby-leaf samples were lower when compared with the inhibition zones recorded for the positive control. 

Also, it seems that Gram-positive isolates are more susceptible than the Gram-negative ones, probably due to the different mechanism of defence of these two groups of bacteria. Our results also showed that except for PEITC, pure compounds were insignificant in inhibiting the growth of the bacterial isolates (Table 7). Comparing the antibacterial effects of phytochemicals with those obtained with traditional antibiotics (gentamicin and vancomicin) according to the antimicrobial efficacy only the phytochemical PEITC was more efficient than the other phytochemicals. The PEITC had in general a moderate efficacy (+) efficacy (*p* < 0.05) against both Gram-positive bacteria and Gram-negative bacteria, and strong efficacy (+++) (*p* < 0.05) against the strain *Staphylococcus aureus* MJS241 from a clinical source, when compared with the antibiotics tested (Table 6). It seems that PEITC is very efficient against *S. aureus* and this is very important since this microorganism is associated with skin infections, pneumonia, endocarditis, toxic shock syndrome (TSS), bacteraemia, and sepsis, and is often reported as one of the bacteria with high resistance to classical treatments with antibiotics [19]. Therefore the use of PEITC might be an interesting alternative tool to be used when the classical approach against these bacteria fails.

**Table 6:** Antimicrobial activity of hydrophilic extracts from baby-leaf salads samples at 10 mg.mL^−1^, expressed as average of diameter of inhibition zone (mm) ^1,2,3^.

Isolates	Source	Green lettuce	Red lettuce	Watercress	Rucola	Mixture ^1^	Mixture ^2^	DMSO ^4^	Commercial antibiotic ^5^
*Escherichia coli *MJS260	Clinical	n.i.	n.i.	n.i.	n.i.	n.i.	n.i.	n.i.	15.7 ± 0.3
*Escherichia coli* 006 AZ	Food	n.i.	n.i.	n.i.	n.i.	n.i.	n.i.	n.i.	17.0 ± 0.3
*Escherichia coli*	ATCC 25992	n.i.	n.i.	n.i.	n.i.	n.i.	n.i.	n.i.	17.0 ± 0.3
*Escherichia coli* 1	Animal	n.i.	n.i.	n.i.	n.i.	n.i.	n.i.	n.i.	18.0 ± 0.4
*Pseudomonas aeruginosa*	ATCC 10145	n.i.	n.i.	n.i.	n.i.	n.i.	n.i.	n.i.	19.5 ± 0.5
*Pseudomonas aeruginosa* MJ323	Clinical	n.i.	n.i.	n.i.	n.i.	n.i.	n.i.	n.i.	16.7 ± 0.3
*Enterococcus spp.* A1Sa	Food	n.i.	n.i.	n.i.	n.i.	n.i.	n.i.	n.i.	20.0 ± 0.3
*Enterococcus faecalis* MJS257	Clinical	n.i.	n.i.	n.i.	n.i.	n.i.	n.i.	n.i.	14.7 ± 0.3
*Enterococcus faecalis*	ATCC 29212	n.i.	n.i.	n.i.	n.i.	n.i.	n.i.	n.i.	14.7 ± 0.3
*Enterococcus faecalis* 49	Animal	8.0 ± 0.0 (+)	9.0 ± 0.3 (+)	11.0 ± 0.3 (+)	13.0 ± 0.3 (+)	9.0 ± 0.3 (+)	11.0 ± 0.3 (+)	n.i.	16.0 ± 0.3
*Staphylococcus aureus*	ATCC 13565	n.i.	n.i.	n.i.	n.i.	n.i.	n.i.	n.i.	16.7 ± 0.6
*Staphylococcus aureus* MJS241	Clinical	n.i.	n.i.	n.i.	n.i.	n.i.	n.i.	n.i.	16.3 ± 0.3

^1^ Values are expressed as mean ± SEM (standard error of the mean) of three assays. ^2^ n.i. — no inhibition effect detected. ^3^ In brackets is presented the classification of antibacterial activity: non effective (−) – inhibition halo = 0; moderate efficacy (+) – 0 < inhibition halo < antibiotic inhibition halo; good efficacy (++) – antibiotic inhibition halo < inhibition halo < 2x antibiotic inhibition halo; strong efficacy (+++) – inhibition halo > 2× antibiotic inhibition halo. ^4^ DMSO — used as negative control. ^5^ Commercial antibiotics used as positive control. Antibiotics used: gentamicin (10 µg disc^−1^) for Gram-negative bacteria and vancomycin (10 µg disc^−1^) for Gram-positive bacteria.

**Table 7:** Antimicrobial activity of pure compounds from baby-leaf salads samples at 10 mg.mL^−1^ and 3.0 µmoles.disc^−1^ (PEITC), expressed as average of diameter of inhibition zone (mm) ^1,2,3^.

Isolates	Source	Gallic acid	Chlorogenic acid	Caffeic acid	Quercitin-3-*O*-rutinoside	Quercitin-3-*O*-rhamnoside	PEITC
*Escherichia coli* MJS260	Clinical	n.i.	n.i.	n.i.	n.i.	n.i.	9.7 ± 0.7 (+)
*Escherichia coli* 006 AZ	Food	n.i.	n.i.	n.i.	n.i.	n.i.	n.i.
*Escherichia coli*	ATCC 25992	n.i.	n.i.	n.i.	n.i.	n.i.	10.0 ± 0.0 (+)
*Escherichia coli* 1	Animal	n.i.	n.i.	n.i.	n.i.	n.i.	11.0 ± 0.0 (+)
*Pseudomonas aeruginosa*	ATCC 10145	n.i.	n.i.	n.i.	n.i.	n.i.	n.i.
*Pseudomonas aeruginosa* MJ323	Clinical	n.i.	n.i.	n.i.	n.i.	n.i.	7.3 ± 0.3 (+)
*Enterococcus spp.* A1Sa	Food	n.i.	n.i.	n.i.	n.i.	n.i.	n.i.
*Enterococcus faecalis* MJS257	Clinical	n.i.	n.i.	n.i.	n.i.	n.i.	14.3 ± 0.3 (+)
*Enterococcus faecalis*	ATCC 29212	n.i.	n.i.	n.i.	n.i.	n.i.	n.i.
*Enterococcus faecalis* 49	Animal	8.0 ± 0.0 (+)	9.0 ± 0.3 (+)	11.0 ± 0.3 (+)	13.0 ± 0.3 (+)	9.0 ± 0.3 (+)	11.0 ± 0.3 (+)
*Staphylococcus aureus*	ATCC 13565	n.i.	n.i.	n.i.	n.i.	n.i.	t.i (+++)
*Staphylococcus aureus *MJS241	Clinical	n.i.	n.i.	n.i.	n.i.	n.i.	44.0 ± 0.0 (+++)

^1^ Values are expressed as mean ± SEM (standard error of the mean) of three assays. ^2^ n.i. — no inhibition effect detected; t..i. — total inhibition. ^3^ In brackets is presented the classification of antibacterial activity: non effective (−) – inhibition halo = 0; moderate efficacy (+) – 0 < inhibition halo < antibiotic inhibition halo; good efficacy (++) – antibiotic inhibition halo < inhibition halo < 2× antibiotic inhibition halo; strong efficacy (+++) – inhibition halo > 2× antibiotic inhibition halo.

The hydrophilic extracts of plants such as extracts of *N. officinale* can be used as easily accessible sources of natural antimicrobial agents due to the presence of PEITC. AS for its antiradical potential, *N. officinale* could be used in the prevention of free radical diseases. Except for *E. faecalis*, we did not find any antibacterial activity for the different hydrophilic plant extracts. The difference of results achieved could have several explanations. The bacterial isolates, the concentrations, the solvents used in extraction, and perhaps local environmental factors such as soil, temperatures, precipitation, and water-stress could affect the biological activity of baby-leaf salads. Thus, further studies are needed with other concentrations, other solvents, other bacterial isolates and probably more compounds.

## 3. Experimental

### 3.1. Plant Material

Thirty fresh baby-leaf species including green and red lettuce (*Lactuca sativa* Var. *longifolia* cv. *romana*; *Lactuca sativa* Var. *crispa* cv. crespa roxa, respectively), rucola (*Eruca sativa* (syn. *E. vesicaria* subsp. *sativa* (Miller) Thell., *Brassica eruca* L.), watercress (*Nasturtium officinale* R. Br., *Brassicaceae*) were purchased from local producers in Vila Real, Northern Portugal. The organic samples were produced with composted manure incorporated in the soil before the sowing of the baby-leaf salads. All the samples were collected at the same physiological age. Two mixtures were prepared in the laboratory; one mixture containing equal proportions (100 g) of green and red lettuce and a second mixture containing equal proportions (25%) of green lettuce, red lettuce, watercress and rucola salad. All samples were freeze-dried (Dura-DryTM μP-FTS Systems, New York, NY, USA), ground and weighed before and after freeze-drying. The respective dry mass was calculated. 

### 3.2. Methods of Sample Preparation for Assessing the Phytochemical Composition, Antioxidant and Antimicrobial Bioassays

All samples were freeze-dried prior to use. Each freeze-dried sample was ground to a fine powder using a blender (model BL41, Waring Commercial, Torrington, CT, USA). The powdered samples were extracted with 70% methanol. 

#### 3.2.1. Methanol Extraction for Total and Individual Phenolics Determination

For methanol extraction, we used the following method: freeze-dried sample (0.5 g) was extracted with 70% aqueous methanol (10 mL) and heated at 70 °C during for 30 min, being agitated (vortex) every 5 min. All extracts were centrifuged (model 2100 Kubota, Tokyo, Japan) at 4,000 rpm during 10 min, filtered (Whatman^TM^ No. 1, 90 mm), and dried in a rotary evaporator at 40 °C until complete dryness, and kept in −20 °C until use in biological assays. Methanolic extracts were used to quantify the total and individual phenolics, to determine the antioxidant capacity and measure the antimicrobial activity.

#### 3.2.2. Determination of Total Phenolic Content

The total phenolic content (TPC) was analysed by Folin-Ciocalteau method, using gallic acid as external standard [20]. Each reaction mixture containing 50 μL of different concentrations of acid gallic or vegetable sample was added to Folin-Ciocalteu reagent (2.5 mL) diluted in bi-distilled water (1:10, v/v) and sodium carbonate (Na2CO3, 2.0 mL), and heated in a water-bath at 45 °C, for 15 min. The blank standard was made replacing gallic acid by 50 μL of methanol (MeOH). The absorbance values of each reacting mixture were measured in U.V. Spectrophotometer (U-2000, serial 121-0120, Hitachi Ltd., Tokyo, Japan) at 765 nm. A gallic acid standard curve was obtained (y = 0.8881x + 0.0233, r² = 0.997) for the calculation of phenolic content. TPC was expressed as mg of gallic acid equivalent (GAE)·100 g^−1^ of dry weight of vegetable sample.

#### 3.2.3. Determination of Individual Phenolics

The quantification of individual phenolics was performed by HPLC analysis, using a HPLC system, equipped with a UV detector, mixing chamber (Gilson-mod. 811A, Middleton, WI, USA), high pressure pump (Gilson-mod. 305); high pressure pump (Gilson-mod. 306), automatic injector (Gilson-mod. 231XL, Middleton, WI, USA); injection unit (Gilson-mod. 402, Middleton, WI, USA); column chamber with temperature controlled (Jones Chromatography, Columbus, OH, USA); reverse phase column (C18 Spherisorb ODS2, 250 mm of length and 4.6 mm diameter). 

For individual phenolics, after sample preparation, 200 µL of each diluted extract were added to HCl (2 M) in 50% aqueous methanol and TBHQ and were placed in a heater at 80 °C for 2 h and then centrifuged (model 2-16 K, Sigma, Osterode, Germany) at 13,000 rpm during 20 minutes. After, the supernatant was removed and was stored at −80°C until its introduction in HPLC system. The eluent was constituted by water with 1% of trifluoroacetic acid (TFA) (solvent A) and acetonitrile with 1% TFA (solvent B). Elution was performed at a flow rate of solvent of 1 mL·min^−1^, with a gradient starting with 100% of water, and the injection volume of 10 μL. The identification was made comparing external standards (Extrasynthese, Lyon, France), their retention times and U.V. The Chromatograms were recorded at 270, 280, 320, and 370 nm for phenolics in general and more specifically 520 nm for anthocyanins.

#### 3.2.4. Determination and Quantification of Individual and Total Glucosinolates

For glucosinolates (GSs), the extraction procedure was performed using the methodology described previously by Pereira *et al.* [21]. Briefly each sample (0.2 g d.w.) was extracted with boiling methanol [90% (v/v), 3 mL] for 2 minutes. A solution of benzyl GLS (glucotropaeolin) 50% (m/v) was used as internal standard. After re-extraction with boiling 70% (v/v) methanol, the supernatant were combined to a final volume of 10 mL. An aliquot (2.5 mL) was evaporated to dryness and resuspended in water (2.5 mL) and 2 mL was applied to a small column of DEAE Sephadex A25 as described previously (Pereira *et al.* [21]). Desulpho-GLS were obtained using commercial aryl sulphatase (EC3.1.6.1) Type H1 from Helix pomatia (Sigma Chemical Co., St. Louis, MO, USA) at 14.6 Units g^−1^. The desulpho-GLS were eluted with water and analyzed using high performance liquid chromatography (HPLC). The procedure adopted corresponds to the ISO 9167-1 method (EEC Regulation No. 9167-1, 1992). GLS peak identification and quantitative estimations were made using pure standard GLS as internal standard (benzyl GLS), and response factors (GLS concentrations were expressed in μmol·g^−1^ dry weight (d.w.). All reagents were of analytical or HPLC grade. The mobile phase consisted of two solvents A and B, being solvent A composed of ultra-pure water and solvent B by acetonitrile (CH_3_CN) at 20%. Elution was performed at a flow rate of 1.5 mL·min^−1^. The chromatograms were recorded at 229 nm and were used to identify glucosinolates (GSs) in vegetable samples. The identification was based on nine external standards. After identification, the GSs were quantified using the following formula:

μmol of GS/100 g*DW = AG/Api*FR*Cpi*100/DW (μmol of GS*100 g^−1^ DW) (1)

GSs were expressed as 100 g of dry weight, in which GS refers to glucosinolates, AG refers to peak area of each glucosinolate to quantify, Api refers to peak area of the internal standard (glucotropaeolin), FR refers to response factor of each GSs identified, Cpi refers to the concentration of the internal standard (glucotropaeolin), Dw refers to dry weight of each sample used in the extraction. This methodology was only performed on rucola and watercress extracts, since GSs are only present in these two types of samples. 

### 3.3. Antioxidant Activity Assays

#### Scavenging of 2,2-Diphenyl-2-picrylhydrazyl (DPPH) Radicals

The free radical scavenging activity of methanolic extracts was measured by the DPPH method [22]. Briefly, different concentrations (0.0 to 5.0 mg.mL^−1^) of methanolic extracts were prepared, diluting the extracts previously obtained in 70% aqueous methanol (v/v). After, methanolic extracts (500 µL) were mixed with a methanolic solution (3.5 mL) containing DPPH• radicals (6 × 10^−5^M in 95% methanol). This mixture was agitated (vortexed) and kept in the dark up to 1 h, in order to obtain stable absorbance values. Then, the free radical scavenging of DPPH• was evaluated by measuring the absorbance at 517 nm, on a U.V. spectrophotometer (U-2000, serial 121-0120. Hitachi Ltd., Tokyo, Japan). The antioxidant activity of each extract was expressed as (%) DPPH scavenging activity, accordingly to the equation: (%) scavenging activity = [(Absblank – Abssample / Absblank] × 100, where A_DPPH_ is the absorbance of free radical DPPH solution and A_sample_ is the absorbance of the solution containing each extract. The compound Trolox ((±)-6-hydroxy-2,5,7,8-tetramethylchromane-2-carboxylic acid, Sigma, Taufkirchen, Germany) was used as positive control. 

For each extract a dose response curve was plotted to determine the EC_50_ values (mg·mL^−1^). EC_50_ is defined as the concentration sufficient to obtain 50% of a maximum scavenging capacity. The experiment was carried out in triplicate.

### 3.4. Antimicrobial Activity *in Vitro* Assays

#### 3.4.1. Microorganisms and Culture Conditions

The bacteria isolates employed in the antimicrobial activity assays are listed in Table 8. These isolates were selected based on their pathogenic importance, since they are associated with human diseases and foodborne disease outbreaks. The bacteria strains were grown from pure cultures preserved in Brain Heart Agar and incubated at 37 °C in order to obtain fresh cultures for the *in vitro* tests.

**Table 8:** Bacterial strains tested in the antibacterial bioassays.

Bacterial strain	Source	Class
*Escherichia coli* MJS260	Clinical	Gram-negative
*Escherichia coli* 006 AZ	Food	
*Escherichia coli*	ATCC 25992	
*Escherichia coli* 1	Animal	
*Pseudomonas aeruginosa*	ATCC 10145	
*Pseudomonas aeruginosa* MJS323	Clinical	
*Enterococcus* spp. A1Sa	Food	Gram-positive
*Enterococcus faecalis* MJS257	Clinical	
*Enterococcus faecalis*	ATCC 29212	
*Enterococcus faecali*s 49	Animal	
*Staphylococcus aureus*	ATCC 13565	
*Staphylococcus aureus* MJS241	Clinical	

##### 3.4.1.1. Antibacterial Susceptibility Tests

For the antimicrobial assay hydrophilic (methanolic) extracts and pure compound (standards) analogues of the bioactive compounds quantified in each leafy extract were used. As positive controls gentamycin (10 µg.disc^−1^) (Oxoid, Basingstoke, Hampshire, UK) for Gram-negative and vancomycin (10 µg.disc^−1^) (Oxoid) for Gram-positive bacteria were used. The test of susceptibility was performed using the disk diffusion on Mueller-Hinton agar plates (Oxoid), accordingly to CLSI procedures [23]. This methodology is based on the principle of diffusion through agar, of the compound with potential antimicrobial properties deposited on a sterile filter paper disc (Oxoid CT0998B). 

##### 3.4.1.2. Antibacterial Classification

The antibacterial effects of the tested methanolic extracts, pure compounds and PEITC were classified according to the following scheme: non effective (−) – inhibition halo = 0; moderate efficacy (+) – 0 < inhibition halo < antibiotic inhibition halo; good efficacy (++) – antibiotic inhibition halo < inhibition halo < 2× antibiotic inhibition halo; strong efficacy (+++) – inhibition halo > 2× antibiotic inhibition halo. The occurrence of an antibacterial effect is observed by the formation of an inhibition zone for bacterial growth. Except PEITC, all extracts and pure compounds were applied (15 µL) at a concentration of 10 mg·mL^−1^. The PEITC concentration used was 3 µmoles·disc^−1^. After 24 h of incubation at 37 °C, the inhibition zones in mm were observed and recorded. All antimicrobial experiments were performed in triplicate.

### 3.5. Statistical Analysis and Quality Assurance of the Results

All data were analysed using the software SPSS version 17.0 for Windows (SPSS Inc., Chicago, IL, USA). All experiments were performed in triplicate and the results were presented as the mean ± SEM (standard error of the mean). The data were analysed using One-Way Anova. The differences between the mean values were separated using Duncan’s test, at significance level of *p* < 0.05. Also, the Principal Components Analysis (PCA) and Pearson correlation was used to correlate the presence of bioactive compounds and the antioxidant activity of hydrophilic extracts and also to distinguish or associate extracts in their biological effect.

## 4. Conclusions

In conclusion, it can be stated that the different baby-leaf salads extracts and their respective mixtures present interesting average levels of bioactive compounds and antioxidant activity. They could be considered as an important and alternative source of natural antioxidants in the human diet. On the basis of these results, factors such as phytochemical composition, concentration and chemical structures of the bioactive compounds can interfere with the antimicrobial activity of baby-leaf extracts. Also, the origin of the bacterial isolate tested is critical. The phytochemical PEITC seems to be a promising product for antimicrobial therapy against the tested bacteria. Further studies should be done to search new compounds from baby-leaf salads that exhibit strong antioxidant and antimicrobial activity. Moreover new bacterial isolates and others pathogens must be tested.
